# SOD and CAT as potential preliminary biomarkers for the differential diagnosis of schizophrenia and bipolar disorder in the first episode of psychosis

**DOI:** 10.1192/j.eurpsy.2023.966

**Published:** 2023-07-19

**Authors:** C. Cachán-Vega, E. Antuña, C. García-González, J. C. Bermejo-Millo, F. Baena-Huerta, L. González-Blanco, B. Caballero, I. Vega-Naredo, J. Bobes, M. P. García-Portilla, A. Coto-Montes, Y. Potes

**Affiliations:** 1 Morphology and Cell Biology, University of Oviedo; 2 Instituto de Investigación Sanitaria del Principado de Asturias (ISPA); 3 Institute of Neurosciences of the Principality of Asturias (INEUROPA); 4 University of Oviedo; 5 SESPA / CIBERSAM, Oviedo, Spain

## Abstract

**Introduction:**

Schizophrenia (SCH) and bipolar disorder (BD) are severe mental disorders which lead to psychotic, affective and cognitive symptoms and often cause a progressive functional deterioration of the individual. The current diagnosis of SCH and BD essentially depends on clinical observation that often leads to misdiagnosis and the introduction of non-specific treatments. Therefore, an early detection and intervention are determinant for a better prognosis. Improving outcomes of a First Episode of Psychosis (FEP) depends mainly on the identification of reliable and discriminatory biomarkers between both disorders.

**Objectives:**

Given that oxidative stress has been tightly involved in multiple metal disorders, the major goal of this work was to characterize oxidative alterations in order to identify potential biomarkers which allow the differential diagnosis in an early stage.

**Methods:**

The study was carried out on samples from 49 subjects (14 women and 35 men), divided into four groups: a control group of 10 individuals not previously diagnosed with any serious mental disorder, 17 patients who had presented a FEP, 12 patients diagnosed with SCH and 10 patients diagnosed with BP. Biochemical analysis were conducted in erythrocyte fraction to characterize the cellular oxidative damage by measuring lipid peroxidation (LPO) levels and the antioxidant defense system by the evaluation of catalase (CAT) and superoxide dismutase (SOD) activities.

**Results:**

In the present work, we observed a significant increase in LPO levels in both SCH and BD disorders that was not neutralized by the antioxidant defense. It was found that SCH patients, despite exhibiting greater activities of SOD and CAT compared to BD individuals, also showed significantly higher levels of oxidative damage. The differential oxidative profile observed between SCH and BD individuals allowed to perform an individually analysis of patients diagnosed with FEP. Although it was not possible to identify the type of psychotic disorder of all the patients with FEP, the results obtained showed that while several individuals exhibited an oxidative prolife similar to that observed in SCH patients, other individuals presented a prolife very similar to that found in patients with BD.

**Conclusions:**

The current work reveals that LPO is a potential indicator of worse prognosis after being differentially modified in both SCH and BD. Moreover, SOD and CAT have been identified, by presenting an opposite profile between patients with SCH and BD, as potential preliminary biomarkers for a discriminatory diagnosis in an early stage of the disorder.

**Disclosure of Interest:**

None Declared

